# Focused ultrasound-mediated small-molecule delivery to potentiate immune checkpoint blockade in solid tumors

**DOI:** 10.3389/fphar.2023.1169608

**Published:** 2023-04-26

**Authors:** Qiuyu Wu, Yuanhang Xia, Xiaohe Xiong, Xinxing Duan, Xiaoxiao Pang, Fugui Zhang, Song Tang, Junlei Su, Shuqiong Wen, Li Mei, Richard D. Cannon, Ping Ji, Zhanpeng Ou

**Affiliations:** ^1^ Chongqing Municipal Key Laboratory of Oral Biomedical Engineering of Higher Education, Chongqing Medical University, Chongqing, China; ^2^ Chongqing Key Laboratory of Oral Diseases and Biomedical Sciences, Chongqing Medical University, Chongqing, China; ^3^ State Key Laboratory of Ultrasound in Medicine and Engineering, College of Biomedical Engineering, Chongqing Medical University, Chongqing, China; ^4^ Department of Oral and Maxillofacial Surgery, Stomatological Hospital of Chongqing Medical University, Chongqing, China; ^5^ Department of Oral Sciences, Sir John Walsh Research Institute, Faculty of Dentistry, University of Otago, Dunedin, New Zealand

**Keywords:** focused ultrasound, small-molecule delivery systems, microbubbles, nanoparticles, immune checkpoint blockade, solid tumors

## Abstract

In the last decade, immune checkpoint blockade (ICB) has revolutionized the standard of treatment for solid tumors. Despite success in several immunogenic tumor types evidenced by improved survival, ICB remains largely unresponsive, especially in “cold tumors” with poor lymphocyte infiltration. In addition, side effects such as immune-related adverse events (irAEs) are also obstacles for the clinical translation of ICB. Recent studies have shown that focused ultrasound (FUS), a non-invasive technology proven to be effective and safe for tumor treatment in clinical settings, could boost the therapeutic effect of ICB while alleviating the potential side effects. Most importantly, the application of FUS to ultrasound-sensitive small particles, such as microbubbles (MBs) or nanoparticles (NPs), allows for precise delivery and release of genetic materials, catalysts and chemotherapeutic agents to tumor sites, thus enhancing the anti-tumor effects of ICB while minimizing toxicity. In this review, we provide an updated overview of the progress made in recent years concerning ICB therapy assisted by FUS-controlled small-molecule delivery systems. We highlight the value of different FUS-augmented small-molecules delivery systems to ICB and describe the synergetic effects and underlying mechanisms of these combination strategies. Furthermore, we discuss the limitations of the current strategies and the possible ways that FUS-mediated small-molecule delivery systems could boost novel personalized ICB treatments for solid tumors.

## 1 Introduction

Cancers are devastating life-threatening diseases. Globally, an estimated 19.3 million new cancer cases, and nearly 10 million cancer deaths, occurred in 2020. By 2040, the global cancer burden is expected to reach 28.4 million cases (Sung et al., 2021). Surgery, chemotherapy and radiotherapy are the major therapies for malignant tumors (Wan et al., 2016), yet frequently they fail to cure a range of cancers, especially solid tumors because of post-surgical tumor residue, resistance to radiotherapy and chemotherapy, or the broad spectrum functional status of the tumor microenvironment (TME) (Xiao and Yu, 2021; Zhang C. et al., 2022). In recent years, the emergence and development of immunotherapy has changed the paradigm of cancer therapy (Mellman et al., 2011; Rosenberg, 2014; Riley et al., 2019). Immunotherapy activates and enhances the anti-tumor immune response, as well as alleviating tumor escape by manipulating the patient’s autoimmune system (Abbott and Ustoyev, 2019; Riley et al., 2019; Kennedy and Salama, 2020). In a variety of experiments and clinical studies, it has been found that immunotherapy has advantages that traditional anti-tumor therapy cannot match. These advantages include: 1) high accuracy, specificity and targeting (Salgia et al., 2003; Schanda et al., 2021); 2) wide adaptability that can control and kill multiple types of tumors (Schmid et al., 2018; Zhang and Zhang, 2020); 3) thorough removal of residual tumor cells (Tan S. et al., 2020); 4) sustained clinical responses to the immunotherapy (Forde et al., 2018; Zhang and Zhang, 2020); and 5) fewer side effects than the traditional treatments (Tan S. et al., 2020). At present, there are approximately a dozen different kinds of immunotherapies for cancer treatment, including immune checkpoint inhibitors (ICIs) that cause immune checkpoint blockade (ICB), lymphocyte-activating cytokines, chimeric antigen receptor (CAR)-T cells or other cellular therapies, agonistic antibodies against co-stimulatory receptors, bispecific antibodies, tumor vaccines and oncolytic viruses (Dillman, 2011; Riley et al., 2019; Ou et al., 2022). Among these strategies, ICB is one of the most well-studied and promising immunotherapies (Webb et al., 2018) because it often has a broader applicability across cancer types and results in a more durable clinical response than other strategies when treatment is effective (Ribas and Wolchok, 2018; He and Xu, 2020). Despite the significant advances achieved, the clinical application of ICB has been impeded by multiple challenges, both in terms of effectiveness (Maleki Vareki et al., 2017) and safety (June et al., 2017). The physical and chemical barriers in solid tumors greatly hinder the entry of therapeutic agents, and this may be responsible for the low response rate in some patients (Kruger et al., 2019). Additionally, the autoimmune side effects caused by systemic delivery of ICB could be dangerous and difficult to overcome (Morad et al., 2021). These problems require novel approaches to administer ICB in a more effective and controllable manner. Advances in delivery technologies, in particular, could improve the curative potential of ICB and could also reduce ICB-related toxicities (Kubli et al., 2021). Multiple delivery modes, including photons (Cheng H. et al., 2022; Chen et al., 2022), ultrasound (Lee H. et al., 2022), alternating magnetic fields (Ge et al., 2023), and radiofrequencies (Shou et al., 2022), can increase the accumulation of small-molecule drugs within the TME, enable more efficient and precise targeting of the specific tumor and/or immune cells, re-shape the TME and reduce off-target adverse effects (Izci et al., 2021). Therefore, combining ICB with various delivery platforms has been one of the promising strategies to improve ICB for solid tumors (Gong et al., 2021).

Among these combination strategies, the recently much-discussed technology, focused ultrasound (FUS), has been found to enhance the anti-tumor immune response in humans (Quaia et al., 2006; Mahnken et al., 2013). Sonodynamic therapy (SDT), which is an application of FUS (Mess et al., 2023), refers to sonochemical or sonophotochemical events in the acoustic field that result in cytotoxicity dependent on the presence of a sensitizer (Xu et al., 2023). It can promote the killing of tumor cells by various mechanisms, including ROS generation and ultrasonic cavitation (Guo et al., 2022). However, such therapy is usually associated with inertial cavitation and unnecessary healthy tissue damage due to the insufficient sensitivity of current sonosensitizers to ultrasound (Fan et al., 2023). In addition to its use in SDT, FUS also excels in the field of treatment through its thermal and mechanical effects (Sivaramakrishnan and Incharoensakdi, 2018; Baek et al., 2021). FUS has the potential to enhance the therapeutic effect of ICB while simultaneously alleviating the potential side effects in solid tumors (Wu et al., 2022). More importantly, FUS-assisted drug deliver is a promising application in solid tumors because it can deliver small-molecule drugs to targeted deep areas with a high degree of precision and minimize the adverse effects on healthy tissues. Small molecules could also be directly delivered into cells by FUS through a variety of mechanisms (Armulik et al., 2010). Furthermore, the FUS-mediated delivery system can also activate the anti-tumor immune response by delivering genes and antigens into the cancer cells or tissues (Unga and Hashida, 2014) or through other ways.

Due to the great potential of FUS-mediated therapy in boosting immunotherapy especially ICB, in this review we summarize the recent research progress of FUS-controlled small-molecule delivery systems in ICB therapy. In addition, we discuss the possible mechanisms by which FUS-mediated small-molecule delivery systems could facilitate personalized ICB therapy for solid tumors.

## 2 ICB treatment and small-molecule drugs for solid tumors

### 2.1 ICB treatment for solid tumors

Physiologically, immune checkpoints are points in cellular pathways at which key molecules maintain the immune homeostasis and protect healthy tissues from immune attack (Mahoney et al., 2015). To date, multiple immune checkpoint molecules have been discovered, including programmed cell death protein 1 (PD-1), cytotoxic T lymphocyte-associated protein 4 (CTLA-4), lymphocyte activation gene-3 (LAG-3 or CD223), T Cell immunoglobulin-3 (TIM-3), B7-H3 (CD276) and B7-H4 (B7S1, B7x, or Vtcn1) and natural killer group protein 2A (NKG2A) (Marin-Acevedo et al., 2021). The two major classes of immune checkpoint molecules are PD-1/PD-L1 and CTLA-4 (Pardoll, 2012; Granier et al., 2017). PD-1 is an inhibitor of both adaptive and innate immune responses (Ahmadzadeh et al., 2009) that plays an important role in reducing ineffective or harmful immune responses and maintaining immune tolerance (Han et al., 2020; Ma et al., 2021). However, some tumor cells can evade recognition and elimination by T Cells through expressing PD-L1, which binds to PD-1 on T Cells to inhibit their activation (Alsaab et al., 2017; Zhou et al., 2017). Blocking the interaction between PD-1 and PD-L1, therefore, enables T Cell-mediated tumor cell death. Likewise, by blocking the interaction between CTLA-4 and its ligands, T Cells remain active and can recognize and kill tumor cells. ICB has been proved clinically efficient for a range of tumors, including melanoma (Maibach et al., 2020), non-small cell lung cancer (Sul et al., 2016), head and neck cancers (Vos et al., 2021), renal cell cancer (Motzer et al., 2015), Merkel cell cancer (Maniar et al., 2020), Hodgkin’s lymphoma (Tobin et al., 2021), gastric cancer (Zeng et al., 2021), urothelial cancer (Ning et al., 2017), cervical cancer (Peng et al., 2022) and mismatch repair deficient (dMMR)/microsatellite instability high (MSI-H) tumors (Heinhuis et al., 2019). The combination of anti-PD-1 and anti-CTLA-4 monoclonal antibodies was approved as a first-line therapy for patients with unresectable or metastatic melanoma (Larkin et al., 2015; Postow et al., 2015), achieving an objective response rate (ORR) of 59% (Johnson et al., 2022).

The clinical impact of ICB has grown considerably over recent decades yet several challenges remained to be overcome. Notably, the response rate to ICB varies for different types of cancers (Table 1). Results from various clinical studies have shown that the clinical response rates to ICB in melanoma, lung cancer, and renal cell carcinoma can reach as high as 20–30% (Topalian et al., 2012; Brahmer et al., 2015); about one-third of advanced metastatic melanoma patients responded to ICB using anti-PD-1 (Postow et al., 2015; Weber et al., 2015). Other types of cancers, such as breast and bladder cancers, have been reported to respond to ICB monotherapies at a rate of approximately 10%–20% (Tabana et al., 2021; van der Heijden et al., 2021). The underlying factors determining the low response rate or non-responsiveness to ICB are being investigated intensely (Maleki Vareki et al., 2017). It is currently believed that immunological phenotypes in which immune cells are either absent (immune desert) or immune cells fail to properly infiltrate the tumor (immune-excluded tumors) (Ji et al., 2012; Hegde et al., 2016) rarely respond to ICB monotherapy, which may explain the heterogeneity of therapeutic efficacy (Herbst et al., 2014). The hostile TME, a highly complex and dynamic milieu, plays the key role in the formation of these phenotypes, which can result in the lack of tumor-infiltrating T Cells, the abnormal regulation of immune checkpoints on tumor cells or T Cells, and adapted immune resistance (AIR) to ICB (Restifo et al., 2016; Dillman, 2017; Zhang J. et al., 2022; Kim et al., 2022).

**TABLE 1:** Clinical response rate of various tumor types to ICB.

Antibodies	Tumor type	ORR	mPFS	MOS	Ref
Anti-PD-1	Melanoma/Advanced melanoma	28%–45%	4–6.9 months	23–36.9 months	Topalian et al. (2012), Larkin et al. (2015), Ribas et al. (2016), Larkin et al. (2019), Wolchok et al. (2022)
	Advanced non-small-cell lung cancer	14.5%–20%	1.9–3.6 months	8.2–9.2 months	Topalian et al. (2012), Brahmer et al. (2015), Rizvi et al. (2015)
	Advanced renal cell carcinoma	25%–59.3%	4.6–15.1 months	20 months	Topalian et al. (2012), Motzer et al. (2015), Rini et al. (2019)
	Breast cancer	18%–18.5%	1.9–2 months	11.2–12 months	Nanda et al. (2016), Adams et al. (2022)
	Head and neck squamous cell carcinoma	14.6%–23%	2.3–3.4 months	8.4–14.9 months	Burtness et al. (2019), Cohen et al. (2019)
	Hepatocellular carcinoma	14.3%–18.3%	3.0–9.4 months	13.2–15 months	El-Khoueiry et al. (2017), Zhu et al. (2018), Finn et al. (2020b), Kaseb et al. (2022), Kudo et al. (2022)
	Esophageal	11.6%–21.5%	1.7–2.6 months	5.8–10.9 months	Fuchs et al. (2018), Kato et al. (2019), Shah et al. (2019), Kojima et al. (2020)
	Colorectal cancer	33%–48%	13.1–16.5 months	31.4 months	Overman et al. (2017), Andre et al. (2020), Le et al. (2020), O'Malley et al. (2022)
	Classical Hodgkin lymphoma	57%–87%	10–14.7 months	Not reached	Ansell et al. (2015), Younes et al. (2016), Armand et al. (2018), Fedorova et al. (2022)
	Pleural/peritoneal mesothelioma	8%–20%	2.1–5.4 months	10–18 months	Alley et al. (2017), Quispel-Janssen et al. (2018), Fennell et al. (2021), Yap et al. (2021)
Anti–PD-L1	Urothelial carcinoma	14.8%–23%	2.1–2.7 months	7.9–15.9 months	Rosenberg et al. (2016), Balar et al. (2017), Galsky et al. (2020), van der Heijden et al. (2021)
	Breast cancer	24%–53%	6.0–7.5 months	21.0–22.1 months	Schmid et al. (2018), Emens et al. (2019), Schmid et al. (2020), Miles et al. (2021)
	Non-small-cell lung cancer	15%–59%	2.7–8.5 months	12.6–18.6 months	Fehrenbacher et al. (2016), Antonia et al. (2017), Socinski et al. (2018), West et al. (2019)
	Advanced renal cell carcinoma	51.4%–52.5%	13.8 months	19.3 months	Motzer et al. (2019), Tomita et al. (2022)
	Hepatocellular carcinoma	30%–65%	3.4–6.9 months	19.2 months	Finn et al. (2020a), Lee et al. (2020), Ann-Lii Cheng et al. (2022), Cheon et al. (2022)
	Small-cell lung cancer	60.2%–68%	5.1–5.2 months	12.3–13 months	Horn et al. (2018), Paz-Ares et al. (2019)
Anti-CTLA-4	Melanoma/Advanced melanoma	10.8%–38%	2.9–4.7 months	12.3–19.9 months	Larkin et al. (2015), Postow et al. (2015), Weber et al. (2015), Larkin et al. (2019), Wolchok et al. (2022)

ORR: overall or objective response rate; mPFS: median progression-free survival; mOS: median overall survival.

Another difficult problem is that systemic administration of ICB can lead to severe irAEs in numerous organs (Friedman et al., 2016; Naidoo et al., 2017; Elia et al., 2020). Early toxicity (between 1 and 12 weeks after treatment initiation) from both CTLA-4 and PD-1 inhibitors most commonly presents as dermatological effects (Sandigursky and Mor, 2018). Other common types of irAEs include gastrointestinal, hepatic and renal toxicities, vitiligo, rash, arthralgia, pneumonitis, hypothyroidism, and hypophysitis (Khoja et al., 2017; Xu et al., 2018; Ramos-Casals et al., 2020). The severity of irAEs varies, but can be serious, or even deadly in some cases (Helmink et al., 2020). Moreover, the onset of irAE usually does not follow a predictable time-course, with some starting days to weeks after therapy and others months later (Postow et al., 2018; Pauken et al., 2019). In light of the above, novel approaches to increase the response rate and reduce the irAEs of ICB are urgently needed. Combination with other therapies, such as small-molecule drugs, may provide a way to overcome these critical problems.

### 2.2 Small-molecule drugs for solid tumors

Small molecule compounds have been studied as drugs for solid tumors for decades and significant progress has been made in recent years. Over 60 small-molecule drugs were FDA-approved for treating solid tumors from 2011 to 2021, including various kinase or enzyme inhibitors, receptor antagonists, transcription inhibitors and therapeutic radiopharmaceuticals (Sochacka-Cwikla et al., 2022). These agents could control the progression of tumors by dampening the proliferation of cancer cells, suppressing angiogenesis or reconstructing the TME (Belgiovine et al., 2017; Gan et al., 2018) when used alone or in combination with other therapeutic agents. Importantly, the additional effects of small-molecule drugs on immunotherapies, especially ICB, are being evaluated in ongoing clinical trials (Yamaguchi et al., 2022). Small-molecule drugs can directly, or indirectly, modulate immune checkpoints and strengthen the anti-tumor effects of ICIs through disrupting the immune checkpoint interactions (CA-170) (Sasikumar et al., 2021), epigenetic regulation (DNA hypomethylating agents, HDAC inhibitors) (Yang et al., 2014; Gao et al., 2020), transcriptional regulation (EGFR inhibitors (Jiang et al., 2017), PI3K-AKT-mTOR pathway inhibition (Janse van Rensburg et al., 2018; Du et al., 2020), JAK-STAT pathway inhibition (Shen M. et al., 2019) or protein stability modification (poly (ADP-ribose) polymerase (PARP) inhibitor (Wu Z. et al., 2021), or through proteolysis-targeting chimeras (PROTACs)) of the immune checkpoint molecules (Cotton et al., 2021). Besides targeting immune checkpoints on T Cells, small-molecule agents can also target the innate immune system. For example, the stimulator of interferon genes (STING) agonists could activate the antigen-presenting cells (APCs), especially CD8^+^ DCs, which promote T Cell infiltration through cross-presentation (Li A. et al., 2019; Ding et al., 2020; Jiang et al., 2020). In addition, these agents increase the expression of antigen-presenting related molecules, including Tap1, Tap2 and MHC-I, with type-I IFN upregulation, which may improve the tumor immune surveillance (Grabosch et al., 2019). Combination with small-molecule drugs and anti-CTLA4 antibody or anti-PD-L1 antibody have shown successful tumor control both in preclinical models and clinical trials (Hellmann et al., 2017). Additionally, small-molecule immunotherapeutics play a role in a TME-directed approach. The tryptophan-metabolizing enzyme, indoleamine 2,3-dioxygenase 1 (IDO1), is expressed in the TME of multiple tumor types, and mediates the depletion of tryptophan and the increase of kynurenine and other metabolites. IDO1 was shown to promote Treg cells and reduce effector T Cell number and functions (Fujiwara et al., 2022). The selective IDO1 inhibitor, epacadostat, displayed a good overall response rate in a phase 1–2 clinical trial in combination with pembrolizumab (Brochez et al., 2017). Unfortunately, the phase 3 trial did not show clinical benefit when compared with pembrolizumab monotherapy in patients with advanced malignant melanoma (Long et al., 2019). This unsuccessful outcome indicated that the combination strategies are not suitable in at least some tumor types, which may benefit from better delivery systems.

Delivery systems offer numerous benefits over the use of small-molecule drugs alone (Toy and Roy, 2016; Oliva et al., 2017). On the one hand, they can be designed to protect therapeutic agents until they are delivered to the targeted tissue (Aikins et al., 2020). On the other hand, delivery systems can make spatial-, temporal-, and dosage-controlled delivery of therapeutics possible if they are responsive to stimuli such as pH (Knight et al., 2019), light (Tao et al., 2020) or ultrasound, thereby keeping the therapeutic agents inactive until they accumulate within targets (Pan et al., 2018). In this review, we focus on ultrasound-based delivery systems, and discuss their safety and efficacy when combined with ICB treatment of solid tumors.

## 3 Focused ultrasound technology

The exploration of the application of ultrasound in medicine began in the early 20th century in an attempt to detect the presence of tumors in brain tissue based on tissue density (White, 1988). In 1950, Wild (1950) reported the difference in density between cancerous and healthy tissues and the diagnostic values of these findings (Wild and Reid, 1956). Since then, the medical applications of ultrasound have been greatly expanded (Newman and Rozycki, 1998), and it remains one of the major imaging approaches, including for the assessment of cancers. Diagnostic ultrasound is usually delivered at a power of 0.1 W/cm^2^, while higher energy administration is classified as either high intensity (1,000–10,000 W/cm^2^), medium intensity, or low intensity (<3 W/cm^2^) (Xin et al., 2016). High-intensity focused ultrasound (HIFU) was first proposed for therapeutic use in 1942, when Lynn et al. found it caused the destruction of liver tissue and changes in brain structure and behavior in living mice (Lynn et al., 1942). It was not until 1999 that Gelet et al. reported the first clinical study of the use of HIFU for the treatment of local prostate cancer (Gelet et al., 1999). Since then, the application of FUS has expanded to treat a wide variety of diseases (Phenix et al., 2014; Zhang et al., 2014; Wu et al., 2017). Basically, FUS can destroy the targeted tissues (Ter Haar, 2016), increase membrane permeability to enhance the uptake and cytotoxic effects of drugs, resulting in tumor cell death in cancerous tissues (Sasaki et al., 2017; Chowdhury et al., 2020; Sasaki et al., 2022).

### 3.1 Thermal and mechanical effects of focused ultrasound

FUS can induce biological effects within deep tissue in a minimally or non-invasive manner (Lafond et al., 2022). The specific actions of FUS can be divided into two main effects, the thermal and mechanical effects. The thermal effects are generated by the large amplitude, high-duty cycle of the HIFU regime, and occur when the local tissue temperature rises higher than the level causing thermal necrosis (>56°C) (Izadifar et al., 2020). Heating effects will lead to protein denaturation and tissue damage as tissue temperatures rise above 43°C (Pahk, 2021). Usually, temperatures at or above 60°C for 1 s will cause irreversible cell death in most tissues (Zhou, 2011). Coagulative thermal necrosis is the main mechanism of tissue destruction in HIFU thermal therapy (Izadifar et al., 2020). HIFU has the ability to focus acoustic energy into a small volume and generate rapid and spatially confined heating of the target while minimizing the impact on surrounding tissues (Tempany et al., 2011). Combined with diagnostic ultrasound (USgFUS), or preferably MR imaging (MRgFUS) to map the lesioned area (Bachu et al., 2021), HIFU can be precisely applied from the minimal distance. It has been reported that this distance was limited to approximately 10 cells (250–300 μm) when ablating hepatocytes (Ter Haar and Robertson, 1993). The other essential mechanisms of action of FUS are the mechanical effects, including acoustic flow, radiative force and, most importantly, cavitation (stable and inertial). These effects are most often generated by high-pressure and short-duration ultrasound pulses, which achieve high instantaneous intensity without accumulating thermal energy or producing thermal effects (Izadifar et al., 2020). Ultrasound can induce shear stress on nearby tissues, or form jet streams and shock waves, depending largely on its intensity (Yang et al., 2021). Various ultrasound-based therapies rely on mechanical effects, including ultrasound microbubble targeted destruction (UMTD) (Tian et al., 2020), histotripsy (Khokhlova et al., 2015; Bader et al., 2016), lithotripsy (Xu Z. et al., 2021), sonodynamic therapy (Lafond et al., 2019), sonothrombolysis (Kooiman et al., 2020), and blood–brain barrier opening (Beccaria et al., 2020; Bunevicius et al., 2020).

### 3.2 Applications of focused ultrasound

Due to the thermal effects of HIFU, the most commonly explored application is thermal ablation (Ter Haar, 2016). Monitoring the formation of lesions during ablative pulses is extremely important from the perspective of efficacy and safety. MRI- and thermometry-guided FUS are more popular than diagnostic ultrasound-guided FUS because they enable real-time monitoring of lesion formation and tissue temperature (Zhang and Wang, 2010). Since its first application, thermal ablation has become a popular treatment for cancers of the bone, liver, pancreas, breast, and kidney (Shehata, 2012). As a non-invasive method, MR-HIFU treatment is characterized as patient-friendly, with low complication rates, few side effects and is repeatable when necessary (Anneveldt et al., 2021). Integration with MRI makes real-time spatial guidance possible, further improving the safety and therapeutic efficacy (Schmitt et al., 2014).

FUS techniques can also be applied in neuromodulation therapies. Low-intensity focused ultrasound (LIFU) plays a useful role in neuromodulation compared with the ablative effect caused by HIFU (Darrow, 2019). Indeed, LIFU produces a non-thermal mechanical disturbance in voltage-gated ion channels that affects electrical signaling across membranes and therefore regulates the neuronal activity. Furthermore, the neuromodulation effects are not limited to the duration of the LIFU treatment but can last for hours to even days (Harary et al., 2018). Both preclinical and clinical studies have reported the relative safety and efficacy of LIFU for neuromodulation. For example, LIFU was reported to inhibit specific thalamic nuclei and regulate the swine sensory thalamus without affecting nearby nuclei, nor creating any tissue injury or inducing any thermal effects to regulate the swine sensory thalamus (Dallapiazza et al., 2018). Some studies have investigated the application of FUS for neuromodulation in patients. Sanguinetti et al. found that FUS could improve mood and affect the connectivity of neural networks related to emotional regulation (Sanguinetti et al., 2020). In addition, FUS has the potential to treat patients with epilepsy and decrease the seizure frequency (Lee C. C. et al., 2022).

Recently, FUS has been shown to improve the immune response against tumors (Hu et al., 2007; Poon et al., 2021). The underlying mechanisms remain unclear, but it is widely accepted that subcellular debris generated *in situ* by FUS are taken up by dendritic cells (Hu et al., 2005) which then triggers cytotoxic T Cell activation (Xing et al., 2008). Pulsed FUS, or low-intensity FUS, have been shown to drive Th1 inflammation, and stimulate production of localized cell recruitment factors and tumor cell surface immunogenic proteins which increase CD8^+^/T regulatory cell proportions (Maloney et al., 2017). Furthermore, reshaping the hypoxic TME by FUS can enhance the endogenous immune response and also improve the response to radiotherapy and chemotherapy (Wang and Sun, 2002; Barsoum et al., 2014; Noman et al., 2014). LIFU-activated adoptive transfer immune cells also generate good anti-tumor effects (Rezayat and Toostani, 2016). Recent research demonstrated that short-pulsed FUS stimulation can activate the CAR-T Cells at the desired time and location with an inducible CAR cassette under the control of a promoter for the heat-shock protein, which suppressed the tumor growth *in vivo* with satisfying safety (Wu Y. et al., 2021).

More importantly, the potential of FUS to assist drug delivery due to its impacts on membrane permeability has recently been explored (Tian et al., 2020). FUS is able to spatiotemporally activate specific drug delivery particles *via* thermal and/or mechanical effects. To respond to the thermal effects of FUS, the particles that carry small-molecule agents to the specific sites are designed to be temperature-sensitive, with a certain heat or energy threshold for the drug to be released (Dromi et al., 2007; Luo et al., 2017). Futhermore, the particles can act as additional nucleation sites and reduce the cavitation threshold during microbubble (MB) formation, which enhances the mechanical effects of FUS and increases drug unloading through acoustic manipulation at the specific positions (Airan et al., 2017; Khirallah et al., 2019; Chen et al., 2021). These particles can be either organic, such as lipid- or polymer-based, or inorganic, for example, metallic, or a combination of both types (Mai et al., 2021). The application of FUS as delivery technology to boost ICB has been a major novel and promising combination strategy and is discussed further below.

## 4 FUS-mediated delivery systems enhance the efficacy of ICB for solid tumors

FUS has been proven as a strategy to improve tumor responsiveness to ICIs (Sheybani and Price, 2019) (Table 2). Studies have shown that HIFU treatment led to an upregulation of immune checkpoint molecules, such as PD-1, PD-L1 or CD80/86 (ligand of CTLA-4) on tumor cells (Eranki et al., 2020; Abe et al., 2022), dendritic cells (DC) (Hu et al., 2005) or T Cells (Garg et al., 2015), resulting in better control of the tumors. More importantly, FUS has also been applied in drug delivery. In this case, FUS is used to improve the local delivery of therapeutic reagents carried by ultrasound-responsive materials and enhance the anti-tumor efficacy (Timbie et al., 2015; Yildirim et al., 2019). These studies indicated the potential of FUS-mediated combinatorial strategies in solid tumors to improve the efficacy of ICB in anti-tumor immunity and outcome.

**TABLE 2:** Clinical trials of FUS and ICB combination therapy in solid tumors.

Rank	NCT number	Status	Conditions	Phases	FUS form	ICB drug	Combination
1	NCT 05491694	Not yet recruiting	Triple-negative breast cancer	Phase 2	HIFU	Toripalimab	HIFU and Toripalimab + epirubicin + cyclophosphamide × 4 cycles → Toripalimab + carboplatin + nab-paclitaxel IVD × 4 cycles every 3 weeks for 8 doses
2	NCT 04116320	Recruiting	Advanced solid tumors	Phase 1	FUSA	anti-PD-1 antibody	Cohort 1: FUSA therapy and standard of care PD-1 blockade (optional combination with intratumoral poly-ICLC) on day 8
							Cohort 2: FUSA therapy (optional combination with intratumoral poly-ICLC) on day 1
3	NCT 05317858	Recruiting	NSCLC brain metastases	Phase 3	MRgFUS	Pembrolizumab	Experimental arm: Exablate BBBD + Pembrolizumab infusion
							Control Arm: Pembrolizumab infusion
4	NCT04021420	Recruiting	Melanoma brain metastases	Phase 1	LIPU	Nivolumab	SonoCloud^®^ (an implantable ultrasound transducer) BBBD and Nivolumab
				Phase 2			
5	NCT 04819516	Recruiting	Colorectal cancer	Phase 1	HIFU	Toripalimab	HIFU and Toripalimab plus regorafenib

BBBD: blood brain barrier disruption; FUS, focused ultrasound; FUSA, focused ultrasound ablation therapy; HIFU, high intensity focused ultrasound; ICB, immune checkpoint blockade; LIPU, low intensity pulsed ultrasound; NSCLC, non-small-cell lung cancer; MRgFUS, MRI guided focused ultrasound surgery. Data obtained *via* https://clinicaltrials.gov, accessed on 3 February 2023.

### 4.1 FUS enhances the efficacy of ICB for solid tumors

To date, the feasibility of FUS and ICB combinatorial regimens in solid tumors is under preclinical investigation. The study of Abe et al. showed that mechanical-HIFU (M-HIFU) elicited stronger systemic cellular antitumor immunity and suppression of tumor growth than thermal-HIFU (T-HIFU) in murine breast cancer models because M-HIFU induced an inflammatory, M1-biased, macrophage population which contributed to enhanced antitumor immunity in M-HIFU-treated tumors (Abe et al., 2022). However, many M-HIFU-treated mice eventually developed progressive disease and the upregulation of PD-L1 rather than PD-1 and TIM3 on neutrophils, DCs, and macrophages was found in M-HIFU-treated tumors. Concurrent anti-PD-L1 antibody administration activated CD8 T Cells markedly and increased CD8 and NK cell-mediated systemic antitumor immunity, which resulted in superior systemic antitumor immune responses and distant tumor growth suppression. In another study, murine orthotopic pancreatic KPC tumors were treated with both pulsed-HIFU (pHIFU) and anti-CTLA-4/anti-PD-1 antibodies (ICI-treated) (Mouratidis et al., 2021). Increased infiltration of CD8^+^ T Cells in the tumors of both pHIFU and pHIFU + ICI-treated groups was observed compared with the sham-exposed group. Survival of the mice in the pHIFU + ICI-treated group was prolonged compared to both control untreated group and pHIFU or ICI alone groups. The study showed that the balance within the tumor was skewed towards a pro-inflammatory phenotype in pHIFU + ICI-treated mice caused by an acute infiltration of CD8^+^ TILs and CD8^+^ IFNγ+ TILs, raising the ratio of CD8^+^ IFNγ+ TILs to regulatory T Cells, MDSCs and CD4^+^ T Cells while retaining the levels of regulatory T Cells and MDSCs at the levels seen in control mice. A similar protocol was also carried out in a refractory murine neuroblastoma model. M-HIFU increased the expression of PD-L1 on most tumor cells significantly and induced a systemic immune activation of DCs, tumor-infiltrating T Cells, proinflammatory cytokine changes, and damage-associated molecular pattern (DAMP) changes, as well as concurrent downregulation of protumor regulators such as regulatory T Cells, IL10, TGFb, and VEGF-A (Eranki et al., 2020). In this study, M-HIFU combined with αCTLA-4 + αPD-L1 significantly prolonged survival from 0% to 62.5% in a refractory unilateral and bilateral neuroblastoma tumor model. This combination treatment of HIFU and ICB was also found to be adoptively transferable, and this could induce long-term memory responses and slow subsequent *de novo* tumor engraftment (Eranki et al., 2020). Another FUS therapy, histotripsy, was used to treat immunocompetent C57BL/6 mice implanted with poorly immunogenic melanoma or hepatocellular carcinoma tumors (Qu et al., 2020). It was found that histotripsy ablation of tumors significantly inhibited primary tumor growth and abscopal pulmonary metastasis by induction of systemic inflammatory changes and stimulation of potent local intratumoral infiltration of innate and adaptive immune cell populations. When used together with anti-CTLA-4 antibody, histotripsy augmented the efficacy of checkpoint inhibition immunotherapy (Qu et al., 2020).

Overall, the combination of FUS and ICB has been shown to exert a superior anti-tumor effect by inducing a local or systemic inflammatory immune activation, and enhancing the intratumoral infiltration and maturity of innate and adaptive immune cells, as well as concurrently downregulating protumor regulators in different types of solid tumors.

### 4.2 FUS-mediated small-molecule delivery synergizes with ICB in solid tumors

**FIGURE 1:**
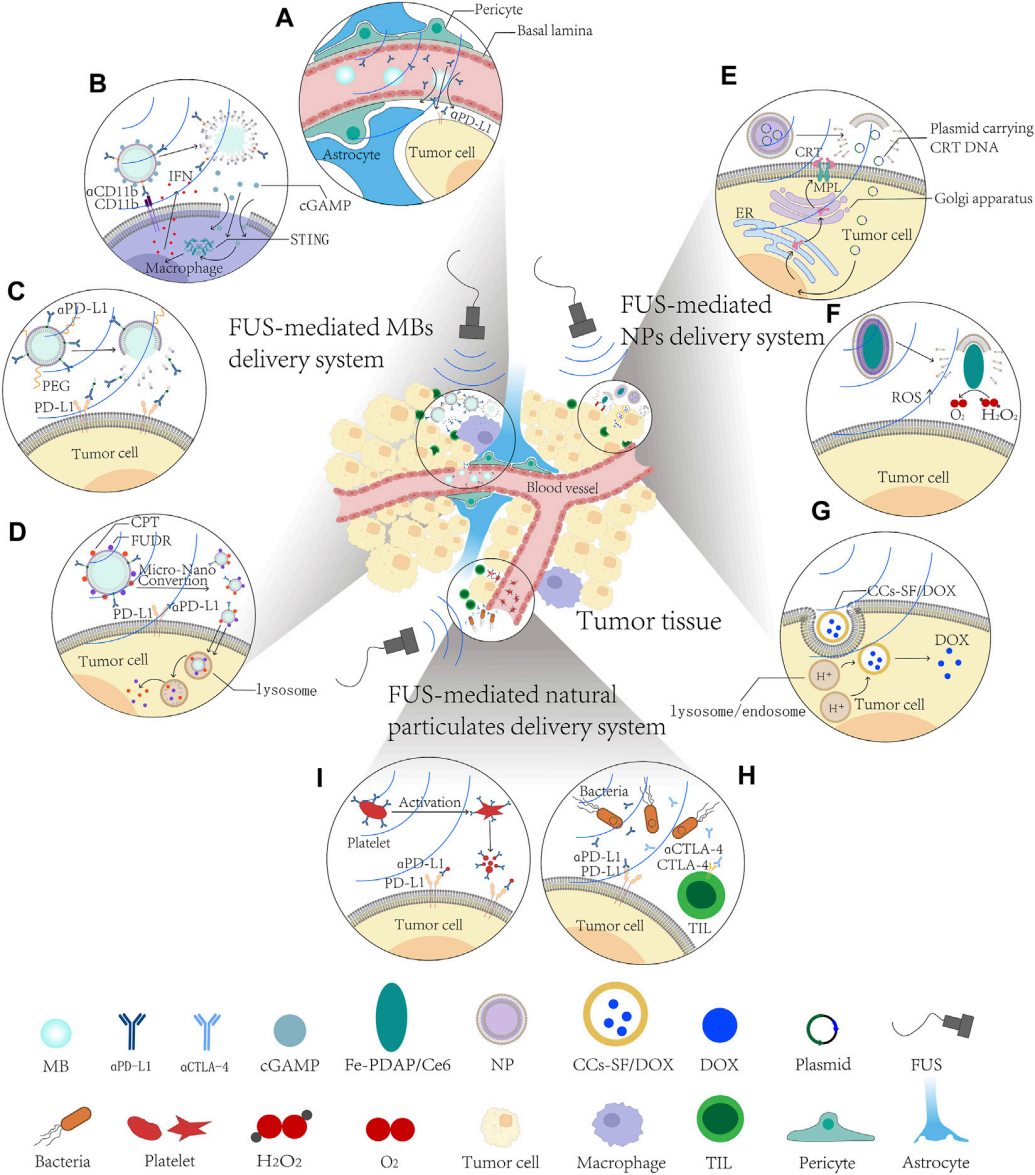
Schematic diagram of FUS-mediated small-molecule delivery systems in solid tumor treatment. **(A–D)** FUS-mediated microbubble delivery system improves the therapeutic effects and diminishes side effects of ICB. **(A)** FUS-MBs delivery system disrupt BBB/BTB to enhance penetration and distribution of αPD1 antibody. **(B)** FUS-mediated cGAMP loading ncMBs targeting macrophages specifically activate cGAS-STING pathway. **(C)** FUS-mediated IMC delivery to alleviate their immunogenicity. **(D)** FUS-mediated αPFC MBs to reach higher tumor cell uptake and deeper tumor penetration. **(E–G)** FUS-mediated nanoparticles delivery system synergizes with ICB in solid tumors. **(E)** FUS-mediated CRT-NPs upregulate CRT expression and induce ICD in tumor cells. **(F)** FUS-mediated MFC targeting TME to stimulate reactive oxygen species (ROS) production. **(G)** FUS-mediated CCs-SF/DOX NPs prevent premature release and increase intracellular nflux of drug. **(H, I)** FUS-mediated natural particulate delivery targets ICs in solid tumors. **(H)** FUS-mediated engineered platelets effectively target incompletely ablated tumor cells and release αPD-L1 antibody. **(I)** FUS-mediated therapeutic bacteria home to, and engraft in, tumors to chronically release αPD-L1 and αCTLA-4. αCTLA-4, anti-CTLA-4 antibody; αPD-L1, anti-PD-L1 antibody; CCs-SF/DOX, silk fibroin-modified doxorubicin preloaded calcium carbonates; cGAMP, cyclic GMP-AMP; CPT, camptothecin; CRT, calreticulin; ER, endoplasmic reticulum; FUDR, floxuridine; FUS, focused ultrasound; IFN, interferon; MBs, microbubbles; MPL, thrombopoietin receptor; NPs, nanoparticles; PEG, polyethylene glycol; ROS, reactive oxygen species; STING, stimulator of interferon genes; TIL, tumor infiltrating lymphocyte.

Accumulative evidence suggests that FUS has potential in small-molecule drug delivery (Mullick Chowdhury et al., 2017; Al Sawaftah and Husseini, 2020) because of its non-invasiveness, clinical convenience, and spatial-, temporal-, and dosage-controlled delivery with a high degree of precision (Tzu-Yin et al., 2013; Mullick Chowdhury et al., 2016). This is especially beneficial for cancer treatment as it could minimize the adverse effects (Al Sawaftah and Husseini, 2020). Various inorganic or organic materials have been used for targeted drug delivery including inorganic nanoparticles (NPs) (Li et al., 2016), liposomes (Zhao et al., 2015), polymer micelles (Shi et al., 2014), and polymer NPs(Shen X. et al., 2019) for superior anti-tumor effect. We have summarized the research progress for FUS-mediated small-molecule delivery combined with ICB in the treatment of solid tumors treatment in Figure 1.

#### 4.2.1 FUS-mediated microbubble delivery system improves the therapeutic effects, and diminishes the side effects, of ICB

MBs are stabilized gas bubbles with diameters typically 1–4.5 μm (Blomley et al., 2001), that were originally used as vascular contrast agents to improve the acoustic contrast in diagnostic imaging. However, when subjected to incident ultrasound bursts, these stable tiny gas-filled bubbles undergo high frequency vibrations and exert circumferential or shear stress to the surrounding cells (Sheikov et al., 2008; Alonso et al., 2010). Cellular and molecular evidence suggests that these physical effects can promote paracellular transport through temporary reorganization of the tight junctions (Jalali et al., 2010; Shang et al., 2011) and facilitate transcellular passage by vesicle/caveolae transport (Deng et al., 2012; Xia et al., 2012). When the ultrasound beam is focused, these physical changes and functions are localized in the focal region and can lead to a local increase in the vascular permeability for up to 24 h post sonication (Hynynen et al., 2001; Aryal et al., 2014; Meairs, 2015), providing a window for spatio-temporal targeted drug delivery (Aryal et al., 2014). These findings indicate the feasibility of US-responsive MBs to deliver various types of anti-cancer agents, including chemotherapeutics, antibodies, nanoparticle drug conjugates, and viruses (Curley et al., 2017; Schoen et al., 2022). Recently, the impacts of FUS-MB delivery on the immune system and TME in solid tumors are under investigation. Numerous studies have revealed that FUS-MB, delivery systems could remodel the TME (Alkins et al., 2016; Song et al., 2021), including affecting the immune cells and tumor vasculatures (Liu et al., 2022), to enhance the effect of immunotherapy. Sheybani et al. investigated the impact of blood brain/tumor barrier disruption (BBB/BTB-D) by FUS and MBs on innate and adaptive immune responses in an orthotopic murine glioblastoma model (Sheybani et al., 2022) (Figure 1A). They found that FUS did not alter the frequency of CD8^+^ T Cells across evaluated organs significantly, but upregulated checkpoint molecules (PD-1 and TIM-3) on these cells were detected at 1-week post-FUS, suggesting the increased activation of the T Cells. Similarly, the study of Lee et al. indicated that a closed-loop controlled MB-FUS system could lead to a better penetration and distribution of the anti-PD1 antibody in the GL261 glioma mouse model (Lee H. et al., 2022). Their proposed delivery system increased the interaction of proinflammatory macrophages within the PD-1-expressing TME without promoting global inflammation in non-tumor tissue. When compared to anti-PD1-treated mice with no sonication, closed-loop controlled FUS-MBs in combination with anti-PD-1 elicited antitumor immunity and conferred a significant improvement in survival. These results indicated that FUS-MB delivery system in combination with adjunct therapeutic strategies targeting immune checkpoints (such as PD-1, CTLA-4, and TIM-3) may improve the outcomes of anti-tumor therapy.

Depending on the surface-modified antibody, the US-responsive MBs can target different types of cells. Li et al. synthesized cyclic GMP-AMP (cGAMP) loaded nanocomplex-conjugated microbubbles (ncMBs) that target antigen-presenting cells (Li et al., 2022) (APCs, Figure 1B). cGAMP can activate the ‘cyclic GMP-AMP synthase-stimulator of interferon genes’ (cGAS-STING) pathway which plays an essential role in priming tumor-specific cytotoxic T Cells (Cousin et al., 2019; Ilovitsh et al., 2020; Xu N. et al., 2021). However, non-specific activation of the cGAS-STING pathway may cause widespread inflammatory responses (Li Y. et al., 2019; Decout et al., 2021), impeding their clinical application. Li et al. found that the ncMBs targeted APCs and efficiently delivered cGAMP into the macrophages’ cytosol when exposed to ultrasound, resulting in the activation of the cGAS-STING pathway and downstream proinflammatory pathways that efficiently prime antigen-specific T Cells (Li et al., 2022). This bridging of innate and adaptive immunity inhibited tumor growth and prolonged survival in both localized and metastatic murine breast cancer models. Furthermore, the addition of anti-PD-1 antibody exhibited not only enhanced primary tumor control, but also a superior survival benefit with a 76% increase in median survival and approximately 60% decrease in pulmonary metastatic nodules.

Although the concurrent application of FUS and ICB has shown some preclinical promise (Eranki et al., 2020; Mouratidis et al., 2021; Abe et al., 2022), few studies indicate that FUS can directly alleviate the adverse effects of ICB. As mentioned above, the irAEs that are caused by off-target binding and overactivation of the immune system (Michot et al., 2016; Moslehi et al., 2018; Postow et al., 2018) are usually diverse and unpredictable, potentially affecting almost every organ system (Wang et al., 2018). The transportation of active pharmaceutical ingredients in carriers such as MBs has become a common way to enhance the pharmacokinetic/dynamic profiles *in vivo* (Ma and Mumper, 2013). Use of ultrasound-sensitive immune-engineered complexes targeting ICs is an emerging strategy that may improve the tumor response rate while reducing the potential side effects. Kim et al. designed an immune-microbubble complex (IMC), in which PD-L1 antibodies were conjugated with MBs to alleviate their immunogenicity because the polyethylene glycol chains on the MBs surface prevented the macrophages from recognizing and activating an immune response against the PD-L1 antibodies (Kim et al., 2020) (Figure 1C). It turned out that the toxicity was markedly decreased when the IMCs were administered intravenously in the CT26 colon carcinoma mice group compared to that of the group receiving the unmodified PD-L1 antibodies at the same concentrations. When combined with FUS, IMCs exhibited higher efficiency in suppressing the tumor growth than the combination of the PD-L1 antibody with FUS, IMC only, PD-L1 antibody only, and FUS only. Enhanced localization of the PD-L1 antibody was also observed in the IMC + FUS group. These results suggested that upon FUS treatment, the IMCs underwent cavitation to increase extravasation, and eventually broke down to expose the individual antibodies which then bound to the surface of PD-L1-expressing tumors. Apart from ICIs, US-responsive MBs could also deliver other anti-cancer agents, such as chemotherapeutics. Sun et al. fabricated US-responsive MBs consisting of camptothecin (CPT)−floxuridine (FUDR) (CF) conjugate and anti-PD-L1 (αPFC MBs) in order to achieve higher tumor cell uptake and deeper tumor penetration (Sun et al., 2022) (Figure 1D). In the TME, αPFC MBs were hydrolyzed, then CPT and FUDR were further released at a fixed 1:1 molar ratio to induce immunogenic cell death (ICD) to enhance anti-tumor efficacy, while the anti-PD-L1 antibody could block the PD-L1 receptor on the tumor cells to promote cytotoxic T Cell (CTL) infiltration, and thus achieve robust therapeutic effects and reduce the irAEs. These findings highlighted the potential of FUS-MBs delivery targeting ICs to boost the therapeutic efficacy and avoid the side effects of ICB.

#### 4.2.2 FUS-mediated nanoparticle delivery systems synergize with ICB in solid tumors

NPs, which are typically less than 100 nm in at least one dimension, have been found to take advantage of the enhanced permeability and retention (EPR) effect to sustainably deliver small-molecule drugs to solid tumors after intravenous administration (Hewakuruppu et al., 2013; Baetke et al., 2015). However, malformed and highly permeable tumor blood vessels, malfunctioning lymphatics and dense extracellular matrix (ECM) in tumors elevate interstitial fluid pressure (IFP) and create a solid physical barrier for drug delivery, leading to a gathering of NPs in the perivascular space of tumors (Viallard and Larrivee, 2017; Zhan et al., 2018; Jin, 2021). Hence, the delivery of NPs from the circulatory system to the particular cells and intracellular targets of the tumor remains inefficient and can be ineffective. Recent progress in materials science and drug delivery makes the application of spatial-, temporal-, and dosage-controlled nanoparticle drug delivery systems possible, and the requisite characteristics of NPs can be achieved by using stimuli-responsive materials during their synthesis (Amreddy et al., 2018). Ultrasound interaction with NPs induces enhanced drug delivery and can also be used in image-guided delivery. Here we describe the strategies using FUS and NPs to deliver anticancer agents to tumors, and improve the synergistic antitumor efficacy of ICB.

It has been extensively reported that the release of DAMPs such as calreticulin (CRT) in solid tumors can lead to local activation of ICD (Obeid et al., 2007; Panaretakis et al., 2008; Galluzzi et al., 2017). CRT enhances the phagocytosis and immunogenic recognition of dying cancer cells by APCs and improves interactions with tumor-infiltrating leukocytes (Wang et al., 2012; Wang et al., 2017). When tumor cells experience ICD, the immune stimulatory effects of ICD increase the populations of tumor antigen-presenting and CTL cells, enhancing antitumor immune responses (Green et al., 2009; Fucikova et al., 2016; Cui, 2017). However, CRT activation is often inconsistent in most solid tumors and generally is insufficient to generate an effective antitumor immune response (Garg et al., 2015). To find a clinically translatable method for reproducibly inducing CRT expression, a novel liposome-based calreticulin-nanoparticle (CRT-NP), in which DNA encoding full-length calreticulin was encapsulated, was developed (Sethuraman et al., 2020) (Figure 1E). The CRT-NP was intratumorally injected into B16F10 melanoma mice followed by FUS treatment. It was found that CRT-NP plus FUS (CFUS) upregulated the CRT expression, expanded the population of melanoma TRP-2 specific functional CD4^+^ and CD8^+^ T Cells and tumor-suppressing M1 phenotype macrophages, and increased the expression of PD-1 and PD-L1 in T Cells. Moreover, CFUS markedly inhibited primary B16 melanoma growth and prevented tumor growth in distal untreated sites. These results suggested that the potential combination of CFUS and PD-1/PD-L1 blockade may provide a better anti-tumor effect.

Depending on the practical needs, NPs can be designed to target the receptors on the cell or the intracellular target elements, but also specific factors in the TME (Yu et al., 2012). Several studies show that the highly hypoxic TME makes it difficult to generate robust reactive oxygen species (ROS) during cancer treatment, leading to insufficient tumor-associated antigen (TAA) generation and a limited anti-tumor immune effect (Phua et al., 2019; Yu et al., 2019; Yang et al., 2020). As a sonosensitizer, chlorin e6 (Ce6) can stimulate ROS production by the ultrasonic cavitation effect, which directly kills the tumor cells (Rosenthal et al., 2004). Hence, membrane-coated Fe-PDAP/Ce6 (MFC), an H_2_O_2_ economizer was developed for on-demand O_2_ supply and sonosensitizer-mediated ROS production during ultrasound activation was developed. Both *in vitro* and *in vivo* experiments demonstrated that MFC could be effectively accumulated in tumor cells and tumor tissue *via* homologous targeting mechanisms, and ultrasound irradiation promoted MFC dissociation and the exposure of Fe-PDAP for a more robust O_2_ supply (Jiang et al., 2022). After combination with anti-PD-1 antibody (αPD-1) administration, MFC-reinforced ultrasound suppressed both primary and distant tumors more effectively. Survival analysis showed that 80% percent of mice treated with MFC-reinforced ultrasound + αPD-1 survived to day 45, a survival rate more than 2-fold higher than that of other groups. Further exploration of the underlying mechanism of the tumor-specific immunological effect evoked by MFC + US+αPD-1 showed an increased number of mature DCs in the tumor-draining lymph nodes (TDLNs) (Jiang et al., 2022). Moreover, the upregulation and infiltration of CD8^+^ T Cells as well as the downregulation of Tregs in distant tumors caused by MFC + US+αPD-1 also contributed to a strong anti-tumor immunity against both primary tumors and distant tumors (Jiang et al., 2022) (Figure 1F). On the basis of this work, Wu et al. constructed a focused acoustic vortex (FAV) system with a hollow cylindrical focal region, which exhibited a larger focal region compared to conventional FUS of the same frequency (Wu et al., 2022). Then a synergistic therapy based on integrated FAV double combination sequence-regulated phase-transformation nanodroplets containing ce6 (CPDA@PFH) with checkpoint blockade immunotherapy was applied to metastatic murine breast cancer in a mouse model. Their study found that FAV + CPDA@PFH resulted in significantly higher ROS levels than the other groups both *in vitro* and *in vivo*. Treatment with FAV + CPDA@PFH led to higher inhibition of tumor growth than that with traditional FUS + CPDA@PFH(Wu et al., 2022). Moreover, FAV + CPDA@PFH combined with anti-PD-L1 antibodies induced a systemic immune response that not only inhibited the primary and distal 4T1 tumor growth but also suppressed lung metastasis (Wu et al., 2022). In addition, FAV + CPDA@PFH + anti-PD-L1 induced long-term immune memory that effectively inhibited tumor growth and prolonged the survival of mice (Wu et al., 2022).

Although significant progress has been made in the use of a large number of chemotherapeutic agents with good anti-tumor effects in preclinical trials, their efficacy in clinical phase trials remains frustrating (Dias et al., 2021; Leon-Ferre et al., 2021). Therefore, new technology is urgently needed to break through the bottleneck of existing chemotherapy. The combination of chemotherapy and immunotherapy has been regarded as redefining cancer therapy (Song et al., 2018). Tan et al. constructed silk fibroin-modified doxorubicin preloaded calcium carbonates (CCs-SF/DOX) that could prevent premature drug release (Tan M. et al., 2020) (Figure 1G). When accompanied with LIFU, the CCs-SF/DOX nanocomposites showed a markedly increased intracellular influx through acoustic pertubation-facilitated delivery or endocytic uptake, which aided the chemosensitization of the cancer. After targeting to TME, the acidic endosomes (pH 5.0–6.0) or lysosomes (pH 4.0–5.0) in the cancer cells promoted the decomposition of CCs and the release of DOX for efficient cytotoxicity, which suppressed tumor growth in both the 4T1 tumor-bearing nude mouse model and the primary breast tumor-bearing immunogenic Balb/c mouse model. Residual CCs-SF/DOX or uploaded DOX from dead or dying cells were then encapsulated in vesicles which fused with the plasma membrane of cells and was excreted into the extracellular space, leading to the formation of extracellular vesicles (EVs). Then the EVs responded to the acidic environment in the TME and infected neighboring cancer cells repeatedly, thus resulting in deep penetration of the drug-based EV therapy. Furthermore, administration of αPD-1 combined with CCs-SF/DOX plus LIFU showed improved synergistic antitumor efficacy (Tan M. et al., 2020).

Synthetic and biological NPs are multifunctional and able to carry different kinds of immunotherapy reagents, DNA, proteins, cytokines, adjuvants and antigens (Gorbet et al., 2020). They can be used in combination with FUS and ICB to promote the clinical therapeutical efforts. However, combining FUS-NP delivery with ICB treatment is still in its early stages, systems and methods to track the NPs and ICB biodistribution, kinetics and their cargo during FUS need to be further explored.

#### 4.2.3 FUS-mediated natural particulate delivery to target ICs in solid tumors

In addition to synthetic drug vehicles, such as MBs and NPs mentioned above, natural particulates also have the potential to be applied in drug delivery (Yoo et al., 2011). They have characteristics of evasion from the host immune system, a natural tropism that engenders highly selective and efficient entry into target cells, longevity in the host, and varied therapeutic payloads (Yeo et al., 2013). Natural particulate-based delivery systems, such as erythrocytes (Han et al., 2018), immune cells (Chu et al., 2018), stem cells (Yeo et al., 2013), viruses (Yeo et al., 2013) and exosomes (Lakhal and Wood, 2011; van den Boorn et al., 2011) have generated increased attention as biocompatible drug delivery systems.

Inspired by the excellent inflammation targeting (Morrell et al., 2014) and immunomodulator releasing ability of platelets (Ruggeri and Mendolicchio, 2007), Han et al. synthesized αPD-L1 antibody-coupled engineered platelets, which could effectively target incompletely ablated tumors and inhibit the residual tumor growth and increase the survival rate in triple-negative breast carcinoma (4T1) mouse models with thermal ablation (Han et al., 2019) (TA, Figure 1H). Moreover, upon their activation and dissociation, the engineered platelets could release anti-PD-L1 antibodies as well as pro-inflammatory cytokines. Based on this result, they further tested the targeting ability of the engineered platelets after other physical ablation treatments including HIFU, photodynamic therapy (PDT) and irradiotherapy (RT), and observed a large recruitment of engineered platelets in all ablated areas with potential residual tumors. This result indicated that the platelet-based delivery strategy could be extended to IC-targeted treatment and improve therapeutic effects. In addition, the impaired immune activity of some tumor cores provides a favorable microenvironment for the colonization and growth of certain bacteria, which can reach the tumors after systemic administration (Dang et al., 2001; Leschner et al., 2009; Kang et al., 2020). Meanwhile, a recent study demonstrated that FUS can control the expression of bacterial genes when used with temperature-responsive repressors (Piraner et al., 2017). Capitalizing on these tumor-infiltrating and FUS-stimulated gene expression properties, Abedi et al. engineered FUS-activated therapeutic bacteria (*Escherichia coli* Nissle 1917) the genome of which was optimized to express anti-CTLA-4 and anti-PD-L1 (Abedi et al., 2022) (Figure 1I). In an A20 tumor mouse model, the systemically administrated engineered bacteria were able to home to, and engraft in, tumors and be reliably and chronically activated by a brief non-invasive FUS treatment to release anti-CTLA-4 and anti-PD-L1 and significantly suppress tumor growth.

Since systemic administration of foreign proteins (such as therapeutic bacteria) may increase the risk of inducing a strong immune response (Chinol et al., 1998), and modification of foreign molecules on the membrane of platelets may to some degree induce alloimmunization (Han et al., 2018), future research should focus on solving these problems. It is evident that FUS-mediated natural particulate delivery targeting ICs could act as a new platform to address a wide array of indications in the foreseeable future.

### 4.3 Combination of FUS, ICB and other treatments for solid tumors

In general, “immunologically cold” tumors such as B16F10 melanoma exhibit limited APC functions, leading to an inability to accumulate cytotoxic infiltrating lymphocytes, upregulated PD-L1 expression on tumor cells, and poor response to ICIs in advanced stages, thereby evading antitumor immunity (Lechner et al., 2013; Twyman-Saint Victor et al., 2015). Studies show that anti-CD40 agonist antibody (αCD40) attaches to the CD40 receptor on APCs, activating CD40 signaling pathway as well as the expression of CD80, IL-12, and CCR7. These effects cause efficient APC activity and T cell-based cytotoxic responses (Beatty et al., 2011; Thompson et al., 2015; Long et al., 2016). Resent research has shown that both thermal (Singh et al., 2019; Sethuraman et al., 2020) and mechanical (Qu et al., 2020) FUS can play a role in immune-modulation. Hence, Singh et al. investigated the effect of FUS-based boiling histotripsy (HT) and *insitu* αCD40 combinatorial therapy on ICB refractory murine melanoma (Singh et al., 2021). They found that HT + αCD40 (HT40) upregulated a variety of inflammatory markers associated with phagocytosis, cell adhesion, cytokines, and antigen processing and presentation, and the proliferation rates of the melanoma-specific memory T Cell population in tumors. Consistent with T Cell activation, the checkpoint marker genes (CTLA-4, PD-L1, PD-1, TIM3, and LAG3) were upregulated in αCD40 and HT40 treatment. The following combination of HT40 and ICB therapy (anti-CTLA-4 and anti-PD-L1) exhibited superior inhibition of distant untreated tumors, and prolonged survival rates compared to the control group. These data indicated that HT and *insitu* αCD40 combinatorial therapy reprogrammed immunologically cold tumors and sensitized them to ICB. Thus this approach may be clinically useful for treating “immunologically cold” tumors.

Not all ICB and FUS combination therapies, however, achieve significant results. Sheybani et al. applied thermally ablative FUS in combination with gemcitabine (GEM), a chemotherapy, in murine metastatic TNBC models (Sheybani et al., 2020). The treatment restricted primary tumor outgrowth significantly, improved survival and enhanced immunogenicity by inhibiting myeloid-derived suppressor cell (MDSC) - driven immunosuppression. The addition of anti-PD-1 antibodies did not markedly enhance growth restriction on a FUS + GEM background. The limited effect of PD-1 blockade on this background may be explained partly by the result that a major source of PD-L1 in the TME is MDSC, which has been inhibited by GEM, and the possibility that other mechanisms of resistance to tumor immunity may play a role in the current model system. Future studies could also focus on targeting PD-L1 or other immune checkpoint molecules such as CTLA-4, Tim3, LAG3, or OX-40.

These studies provide timely and provocative insights into the immunogenicity of FUS and the role of FUS-ICB-based combinatorial strategies in solid tumors. To this end, future studies are expected to optimize treatment frequency, and drug dosage in larger animal models and clinical trials.

## 5 Challenges and limitations

Although the field of drug delivery systems as a whole is advancing at a rapid pace, the design of FUS-mediated delivery technologies for this field is still in its infancy, especially when in combination with ICB for human solid tumors. The major challenges still remain in the TME, with high interstitial fluid pressure, compressed vasculature and dense fibrotic tissue surrounding solid tumors that hinders T Cell infiltration and dampens the efficacy of ICB. Small molecule agents that change the physical conditions of TME could be the future targets of the FUS-mediated delivery systems. Losartan (an angiotensin inhibitor), which is under investigation in patients with pancreatic cancer in a phase II clinical trial (NCT01821729), was shown to potentiate the therapeutic delivery of chemotherapeutic agents to several solid tumor types (Chauhan et al., 2013). Treatment with angiotensin inhibitors was reported to normalize the extracellular matrix, reduce the progression of pancreatic cancer, and increase the T Cell and APC activity (Liu et al., 2017), which indicates the possibility of reshaping the TME with the assistance of FUS. In addition, the cellular components in the TME could be another type of fundamental target exploited to deliver immunotherapy. For example, the tumor-associated macrophages (TAMs) could be engineered to directly attack tumor cells by inhibiting SHP substrate 1 (Alvey et al., 2017), or spare the T Cell from exhaustion by losing TAM antigen presentation in an IRF8-dependent manner (Nixon et al., 2022). It is noteworthy that the immune checkpoint molecules, such as PD-1 and Tim-3, are also the markers of exhausted T Cells. The upregulated expression of these markers post-FUS indicated that FUS may affect the T Cell exhaustion program, but the underlying mechanism remains unclear (Garg et al., 2015; Sheybani et al., 2022). Further understanding of the molecular regulation of T Cell exhaustion could improve the designs or technology of FUS-mediated delivery, and provide novel targets to relieve or even reverse the dysfunctional status of cytotoxic immune cells.

Despite its potential, several limitations of FUS-assisted drug delivery remain to be solved, including the short duration of the effect and erratic drug uptake (Sun et al., 2021). In clinical practice, larger tumors may require longer and more complex treatment regimes (Payne et al., 2010), which raises safety concerns. A better understanding of ICB penetration into the tumor and uptake from the circulatory system following different ultrasound schemes and conditions in different kinds of tumor models is needed to optimize treatment protocols. Future work could focus on developing novel technologies to prolong the controllable duration of drug delivery, and more preclinical models and clinical trials are needed to examine their safety.

## 6 Conclusion

ICB has been extensively explored as one of the most promising cancer immunotherapies, yet it has failed to acquire satisfactory clinical outcomes for solid tumors. Small-molecule drugs could assist ICB through different mechanisms, but need precise and safe delivery. In this review, we summarized the current frontline studies of FUS-assisted small-molecule drug delivery in combination with ICB to treat solid tumors. This research provides a proof-of-concept for FUS-mediated delivery serving as a pioneer and effective combinatorial regimen for ICB. By further understanding the underlying mechanisms of the TME and developing novel technologies, FUS-mediated small-molecule delivery could definitely boost the efficacy and the safety of ICB in solid tumors.

## Figures and Tables

**Table:** 

**AIR**	adapted immune resistance
**APC**	antigen-presenting cell
**BBB/BTB-D**	blood brain/tumor barrier disruption
**CAR**	chimeric antigen receptor
**CCs-SF/DOX**	silk fibroin-modified doxorubicin preloaded calcium carbonates
**Ce6**	chlorin e6
**CFUS**	CRT-NP plus FUS
**cGAMP**	cyclic GMP-AMP
**cGAS-STING**	cyclic GMP-AMP synthase-stimulator of interferon genes
**CPT**	camptothecin
**CRT**	calreticulin
**CRT-NP**	calreticulin-nanoparticle
**CTL**	cytotoxic T-lymphocyte
**CTLA-4**	cytotoxic T-lymphocyte-associated protein 4
**DAMP**	damage associated molecular pattern
**DC**	dendritic cell
**dMMR**	mismatch repair deficient
**ECM**	extracellular matrix
**EPR**	enhanced permeability and retention
**ER**	endoplasmic reticulum
**EV**	extracellular vesicle
**FAV**	focused acoustic vortex
**FUDR**	floxuridine
**FUS**	focused ultrasound
**GEM**	gemcitabine
**HIFU**	high intensity focused ultrasound
**HT**	FUS-based boiling histotripsy
**ICB**	immune checkpoint blockade
**ICD**	immunogenic cell death
**ICI**	immune checkpoint inhibitor
**IDO1**	indoleamine 2,3-dioxygenase 1
**IFN**	interferon
**IFP**	interstitial fluid pressure
**IMC**	immune-microbubble complex
**irAE**	immune-related adverse event
**LAG-3**	lymphocyte activation gene-3
**LIFU**	low intensity focused ultrasound
**MB**	microbubble
**MDSC**	myeloid-derived suppressor cells
**MFC**	membrane-coated Fe-PDAP/Ce6
**M-HIFU**	mechanical-HIFU
**mOS**	median overall survival
**mPFS**	median progression-free survival
**MSI-H**	microsatellite instability high
**ncMB**	nanocomplex-conjugated microbubble
**NKG2A**	natural killer group protein 2A
**NP**	nanoparticle
**ORR**	overall or objective response rate
**PD-1**	programmed cell death protein 1
**PD-L1**	programmed cell death ligand 1
**PDT**	photodynamic therapy
**PEG**	polyethylene glycol
**pHIFU**	pulsed-HIFU
**PROTAC**	proteolysis-targeting chimera
**ROS**	reactive oxygen species
**RT**	radiotherapy
**SDT**	sonodynamic therapy
**STING**	stimulator of interferon genes
**TA**	thermal ablation
**TAA**	tumor-associated antigen
**TAM**	tumor-associated macrophage
**TDL**	tumor-draining lymph node
**T-HIFU**	thermal-HIFU
**TIL**	tumor infiltrating lymphocyte
**TIM-3**	T Cell immunoglobulin-3
**TME**	tumor microenvironment
**UMTD**	ultrasound microbubble targeted destruction
